# Glassy states and super-relaxation in populations of coupled phase oscillators

**DOI:** 10.1038/ncomms5118

**Published:** 2014-06-20

**Authors:** D. Iatsenko, P.V.E. McClintock, A. Stefanovska

**Affiliations:** 1 Department of Physics, Lancaster University, Lancaster LA1 4YB, UK

## Abstract

Large networks of coupled oscillators appear in many branches of science, so that the kinds of phenomena they exhibit are not only of intrinsic interest but also of very wide importance. In 1975, Kuramoto proposed an analytically tractable model to describe these systems, which has since been successfully applied in many contexts and remains a subject of intensive research. Some related problems, however, remain unclarified for decades, such as the existence and properties of the oscillator glass state. Here we present a detailed analysis of a very general form of the Kuramoto model. In particular, we find the conditions when it can exhibit glassy behaviour, which represents a kind of synchronous disorder in the present case. Furthermore, we discover a new and intriguing phenomenon that we refer to as super-relaxation where the oscillators feel no interaction at all while relaxing to incoherence. Our findings offer the possibility of creating glassy states and observing super-relaxation in real systems.

## Abstract (web summary)

The Kuramoto model attempts to describe the behaviour and properties of networks of coupled oscillators. By studying a generalization of the original Kuramoto model, Latsenko *et al.* identify several previously unseen complex phenomena that can appear in such networks.

## Graphical abstract

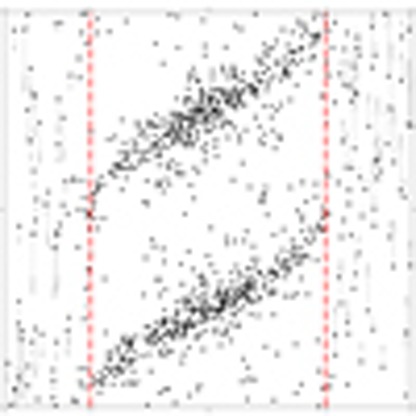

The Kuramoto model (KM)^1^ was introduced and developed to provide an analytically tractable description of the populations of coupled phase oscillators that so often appear in real life. It has been applied successfully in many fields^2^,^3^, for example, to describe the collective behaviour of lasers^4^,^5^, neurons in the brain^6^,^7^, Josephson junction arrays^8^ and even humans^9^.

The widespread applications of the KM to real problems has ensured that the basic model, together with its variants and modifications^10^,^11^,^12^,^13^,^14^,^15^,^16^,^17^,^18^,^19^,^20^,^21^,^22^,^23^,^24^, has been studied very extensively over the past few decades. The practical usefulness of these studies stems from widespread abundance of user-defined systems that can be described by the KM, for example, the above-mentioned laser arrays^4^,^5^ and Josephson junction circuits^8^, where one can adjust the coupling between the oscillators quite freely. Therefore, if some new behaviour has been uncovered theoretically, it can immediately be implemented and observed in practice. Furthermore, for those systems where the exact configuration is not known precisely and cannot be changed, such as interacting neurons in the brain^6^,^7^, it is obviously useful to know what kinds of dynamics the different forms of KM can demonstrate, so that the observed behaviour can be matched with an appropriate existing model and thus explained.

The KM has been found to exhibit a remarkable diversity of interesting phenomena and states and, by now, most of them have been thoroughly investigated. There is, however, one important exception—the so-called ‘oscillator glass’ state^25^. Thus, many systems, including networks of interacting spins^26^,^27^, dipoles^28^, and electrons^29^, have been found to display behaviour reminiscent of a glass structure^30^, and it has been suggested^25^ that populations of coupled oscillators can also demonstrate glassy states of some kind. Studies of such states are expected to be very fruitful, in the same way as were the studies of spin glasses, which led to many new techniques and applications in other fields (biology, computer science, economics and so on^27^). However, the provenance and properties of the oscillator glasses are still subject to debate^25^,^31^,^32^,^33^,^34^,^35^,^36^.

Here we report the analysis of a very general, yet analytically tractable, form of KM. We derive a set of equations describing its steady-state behaviour and show that, for a particular class of distributions, the consideration may be reduced to a much simpler model. Thus, in the limit *t*→∞, the system can demonstrate the same macroscopic behaviour for different coupling configurations, enabling one to select and study the simplest one. This is, in itself, an interesting theoretical result. It also implies that there may be cases where it is, in principle, impossible to infer the underlying coupling structure from the observed data.

We also discuss when and how the full-time evolution of the system parameters can be obtained and find some interesting features related to this question. Among the phenomena that the model can exhibit, we find states with a glassy structure that can be studied analytically within the framework presented, and we discuss these states and their properties in detail. Finally, we describe a completely new phenomenon which we refer to as super-relaxation where, under certain conditions, the coupling between the oscillators effectively disappears during their relaxation to incoherence.

## Results

### Outline of the results

To make what follows clearer and easier to understand, we start by summarizing the main results of the work. First, we derive equations (18, 19, 20, 21 describing the stationary states (SSs) of the system (1). Then we reduce the macroscopic steady-state behaviour of the system (1) (five distributed parameters) to a much simpler model (29) (two distributed parameters) for a large class of distributions (equation (23)). We discover new states in the coupled oscillator populations, which are in some sense similar to physical glasses, and we establish the conditions for their appearance (equations (36) and (37)). And finally, we discover the emergence of a super-relaxation phenomenon, where the oscillators evolve interaction-free, irrespectively of the couplings between them, if the conditions (41), (42), (43) are fulfilled. After reviewing the background and terminology, each of these results is obtained and considered in detail below.

### The model

We consider the KM in the form


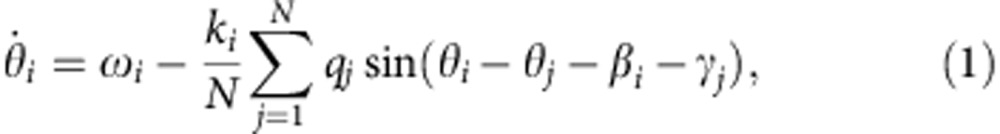


where *N* is the number of oscillators, *θ*_*i*_(*t*) and *ω*_*i*_ are the *i*th oscillator’s phase and natural frequency, respectively, and *k*_*i*_*q*_*j*_ (*β*_*i*_+*γ*_*j*_) represent the coupling strengths (phase lags) between the *i*th and *j*th oscillators; all parameters **Γ**_*i*_≡{*ω*_*i*_, *k*_*i*_, *q*_*i*_, *β*_*i*_, *γ*_*i*_} are drawn from a joint probability density *G*(**Γ**)≡*G*(*ω*, *k*, *q*, *β*, *γ*). The case considered earlier^10^ corresponds to (1) with *q*_*i*_=1, *β*_*i*_=*γ*_*i*_=0.

The KM has not previously been treated in such a general form (1), though it actually includes as special cases the many KM modifications and extensions studied earlier. Thus, not much is known about the possible behaviour of the system except in those particular cases. To study it, we first generalize the recently presented framework^10^ to encompass (1), and then we proceed to a consideration of the phenomena that it can exhibit.

### Main equations

The oscillators’ collective behaviour in (1) can be described by two complex parameters


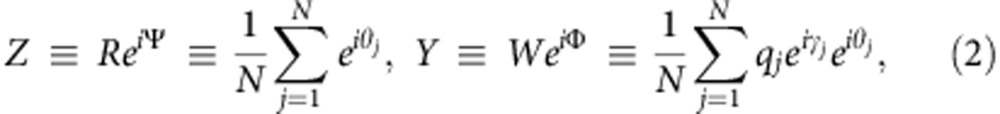


where *Z* is the mean field whose amplitude *R* quantifies the extent of the agreement between the oscillators’ phases *θ*_*i*_, while *Y* represents the weighted mean field, with amplitude *W* reflecting the agreement between *θ*_*i*_+*γ*_*i*_ (+*π* for *q*_*i*_<0), weighted by |*q*_*i*_|. With the use of the definition (2), the model (1) can be rewritten as


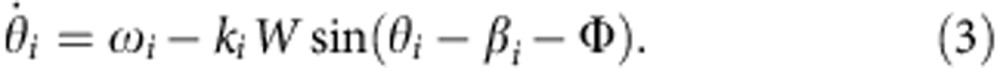


As can be seen, the dynamics of the system is governed mathematically by the weighted mean field *Y*, which determines the effective interaction between the oscillators. The ordinary mean field *Z*, on the other hand, represents a more physical macroscopic variable, being the one that is most often observed in practice (for example, it quantifies the overall output current of a Josephson junction array^8^). Note that, unlike the mean fields’ phases Φ and Ψ individually, their difference Φ−Ψ is invariant under the phase shifts *θ*_*i*_→*θ*_*i*_−*φ*(*t*), and thus represents another meaningful parameter.

In the continuum limit *N*→∞, the system (1) is treated using the probability density function *f*(*θ*,**Γ**,*t*), which reflects the probability that the oscillator has parameters **Γ** and phase *θ* at time *t*. It can be further factorized as


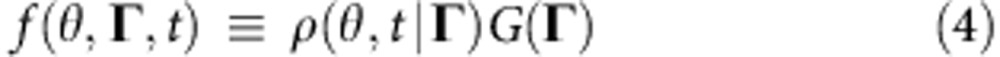


where *ρ*(*θ*,*t*|**Γ**) is the conditional probability density function (CPDF), reflecting the probability that at time *t* the oscillator has a phase *θ*, given its parameters **Γ**. By definition, the CPDF should satisfy 
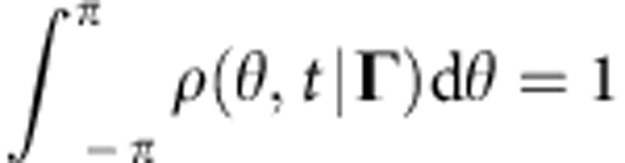
, which leads to the continuity equation


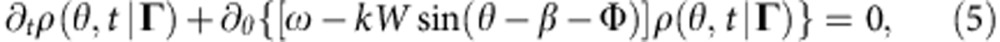


where we have used expression (3) for 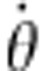.

The CPDF can usually^37^,^38^,^39^,^40^ be represented using the ansatz introduced by Ott and Antonsen (OA-ansatz)^41^


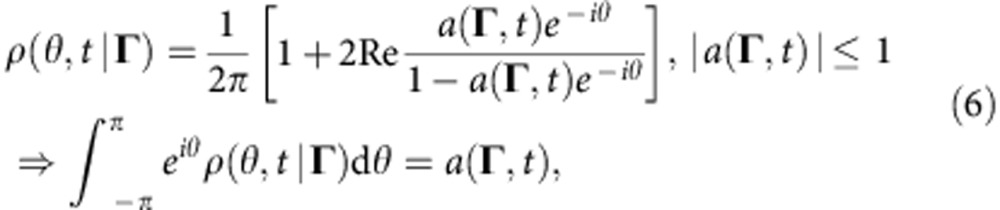


where we use *a*(**Γ**,*t*)=α*(**Γ**,*t*) instead of the original OA-variable^41^ *α*(**Γ**,*t*), and the * denotes complex conjugate.

Finally, by substituting equation (6) into equations (5) and (2), one obtains the full system of equations describing the dynamics of the system (1):


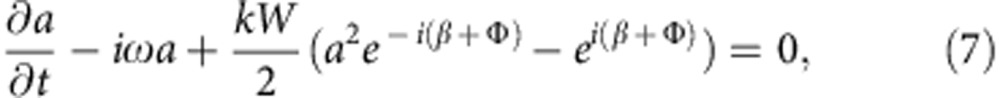



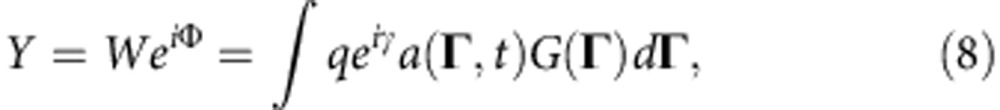



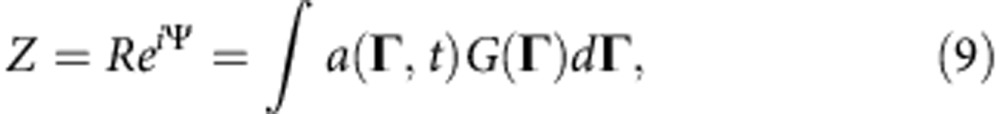


where here and below, unless otherwise specified, the integration over d**Γ**≡d*ω*d*k*d*q*d*β*d*γ* is taken over the whole domain ((−∞, ∞) for *ω*, *k*, *q* and (−*π*, *π*) for *β*, *γ*).

### Parameter redundancy

It should be clarified that parametrizing the KM (1) with both *β*_*i*_ and *γ*_*j*_ is, in a strict sense, mathematically redundant, since one of these phase shifts can be removed by a change of variables. Thus, in terms of 
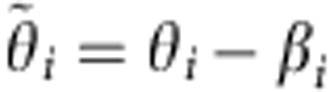
, the system equation (1) becomes


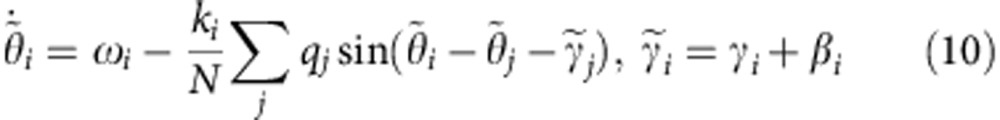


with the new distribution of parameters 
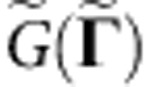
 being given as 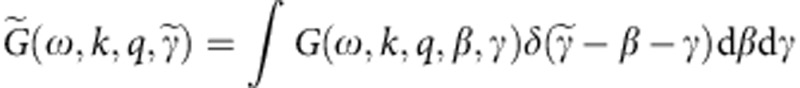. Taking into account the relationship between the variables in equations (10) and (1), it is clear that the conditional distribution of the new phases 
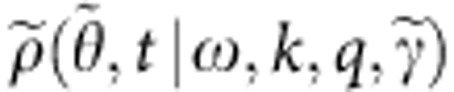
 is related to the original one as 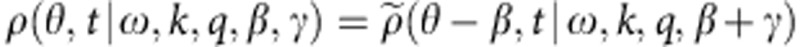. Substituting this into equation (2), it can be shown that the weighted mean fields in terms of *θ*_*i*_ and 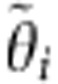 are equal, 
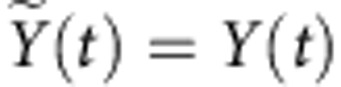
, but that the ordinary mean fields can, in general, change in a non-trivial fashion, 
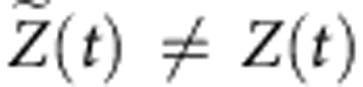
.

Nevertheless, both *β*_*i*_ and *γ*_*j*_ can sometimes be practically relevant, that is, have physical meanings. For example, such a coupling structure can be designed artificially in the case of user-defined systems (for example, laser arrays^4^,^5^), but we expect it to appear in the observation-only systems as well (for example, interacting neurons^6^,^7^). Hence, in some cases, the full model (1) will actually provide a more straightforward and meaningful description of the system, while equation (10) will represent only a mathematical formulation. Thus, for example, *Z* might be related to the real physical quantity, such as the total power output of the laser array^4^,^5^, while 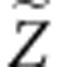 will then be a purely mathematical characteristic. To preserve generality, therefore, and to avoid complicating the discussion by introducing different variable transformations, we study the model (1) including both *β* and *γ*. Furthermore, as will be seen below, the distributed *β* also has significant consequences in terms of the non-equilibrium dynamics, which cannot be straightforwardly obtained from the transformed system equation (10).

It should also be noted, that the model (1) is invariant under (*k*_*i*_,*β*_*i*_)→(−*k*_*i*_,*β*_*i*_+*π*) or (*q*_*i*_,*γ*_*i*_)→(−*q*_*i*_,*γ*_*i*_+*π*) or rescalings (*k*_*i*_,*q*_*i*_)→(*k*_*i*_/*r*,*rq*_*i*_); to preserve generality, we retain this ambiguity, as the normalization can be fixed at any time, and there is no universal choice (see Methods).

### Terminology and notation

We adopt terminology similar to that introduced earlier^10^,^42^. The KM form (1) is invariant under *θ*′=*θ*−Ω*t*, which just changes the natural frequencies to *ω*′=*ω*−Ω, so the parameter distribution becomes *G*′(*ω*′, *k*, *q*, *β*, *γ*)=*G*(*ω*′+Ω, *k*, *q*, *β*, *γ*). Thus, one can consider the KM in different frames rotating at frequency Ω with respect to some reference frame. For the latter, we select the frame with zero mean frequency 〈*ω*〉≡∫*ωG*(**Γ**)d**Γ**=0 and call it the natural frame; the distribution *G*(**Γ**) is defined in this frame. We define a SS as a state with time-independent CPDF ∂_*t*_*ρ*(*θ*,*t*|**Γ**)=0, which also implies ∂_*t*_*Z*=∂_*t*_*Y*=0. The state can be stationary only in a particular rotating frame, so it is characterized by its frame frequency Ω and mean fields *Z*,*Y*. We refer to SSs with Ω=0 as natural states, while SSs with Ω≠0 are travelling wave states. For later convenience we define


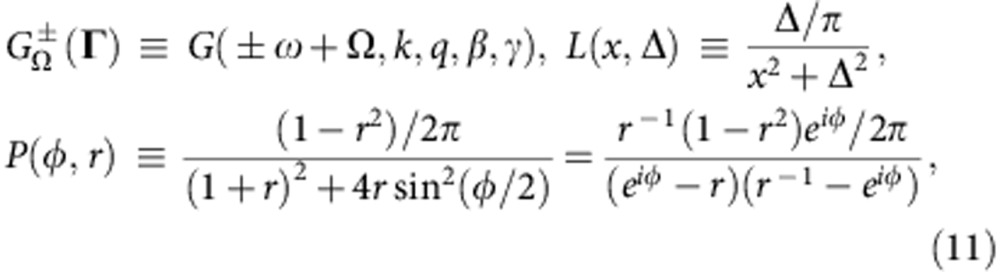


where 0≤*r*≤1; the distribution *P*(*ϕ*,*r*) and the way to simulate it are discussed in Methods. Note, that *P*(*ϕ*,0)=1/2*π* and *P*(*ϕ*,1)=*δ*(*ϕ*).

We also introduce


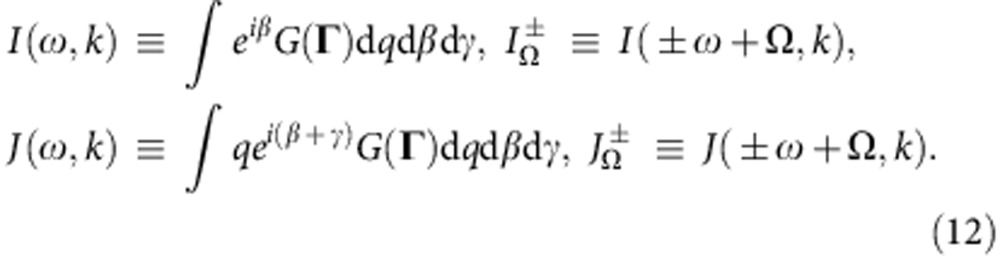


As will be seen below, *I* and *J* represent an effective complex distribution of *ω*,*k* to be used for the determination of *Z* and *Y*, respectively.

### Validity of the OA-ansatz

In what follows, we will make frequent use of the OA-equations (7, 8, 9). For the case *k*_*i*_=const, *q*_*i*_=1, *β*_*i*_=*γ*_*i*_=0 and for a very large class of frequency distributions, their validity has been proven^39^,^40^ in the asymptotic limit *t*→∞, corresponding to system’s steady-state behaviour; though not justified rigorously, this result seems to hold for distributed *k*, *q*, *β*, *γ* as well (based on numerical simulations). As the next step, Pikovsky and Rosenblum^37^,^38^ derived more general equations and showed that the full dynamics of the model—for all *t* and almost any parameter distribution—obeys equations (7)–(9) only if the initial phase configuration also belongs to the OA-manifold


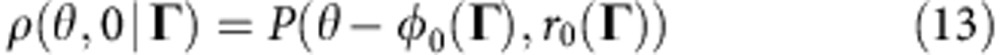


with any *ϕ*_0_(**Γ**) and *r*_0_(**Γ**). Although not given explicitly by Pikovsky and Rosenblum^37^,^38^, equation (13) can be deduced from their equation (3) in ref. 38, which generates (13) in the case of a uniform distribution of the constants of motion *ψ*_*k*_ (for which the OA-description was proven to hold^37^,^38^); see also the work ofMarvel *et al.*^43^ Note that the OA-ansatz often provides a good approximation to the system dynamics even when equation (13) is not satisfied (for example, for *ρ*(*θ*,0|**Γ**)=*R*(0)*δ*(*θ*)+(1−*R*(0))/2*π*).

In addition, it might sometimes be useful to analytically continue *a*(**Γ**,*t*) into the complex plane over some of **Γ**, for example, to consider *ω* to be complex. This is required to apply the conventional OA-reduction procedure^41^ (see also refs 13, 14, 15) which, where possible, allows one to obtain a finite-dimensional system of equations for *Y*(*t*),*Z*(*t*). However, one should always have |*a*(**Γ**,*t*)|≤1 as, otherwise, the OA-ansatz (6) becomes ill-defined. Hence, *a*(**Γ**,*t*) can be considered only in the region of **Γ** for which


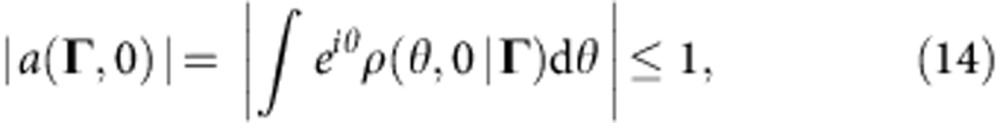



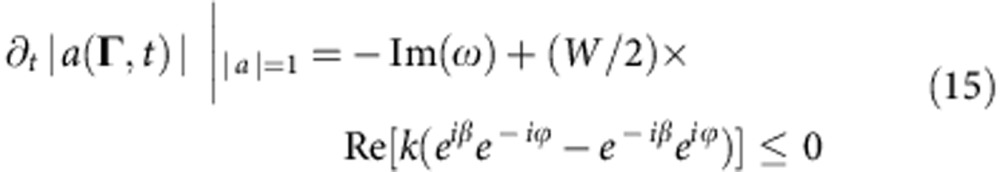


at any *W* and *φ*≡arg[*a*(**Γ**,*t*)]−Φ. The condition (14) establishes that |*a*|≤1 is satisfied at *t*=0, while (15) then guarantees that it holds at all other times too. Some manifestations of the issues related to (14) and (15) can be found in Section 3.2.0.1 of ref. 38 and in refs 11, 13, 14, 15. Note that (14) is always satisfied when there is no correlation of initial phases with the system parameters **Γ**:


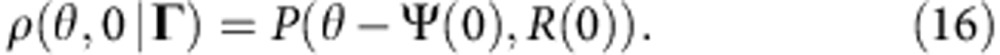


Unless otherwise specified, we will assume that the system starts from such a configuration.

### Stationary states

Having reviewed the background and related issues, we now proceed to the derivations. We start by finding possible configurations into which the system (1) can settle as *t*→∞. Although in specific cases it can converge to some inherently time-dependent solution, such as a standing wave^43^ or an oscillating *π*-state^44^, we restrict the consideration to SSs only, treating them in frames where they are stationary. The effective parameter distribution therefore becomes *G*(**Γ**)→*G*_Ω_^+^(**Γ**), with Ω denoting SS frame frequency. By definition and equation (6), the SSs satisfy ∂_*t*_*a*=0. Using this in equation (7) and taking account of the OA validity condition |*a*|≤1, one finds that all possible SSs in their own rotating frames correspond to


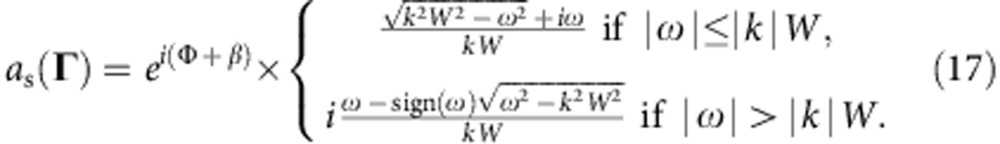


The stationary distribution of the oscillators’ phases is then recovered by substituting equation (17) into equation (6), which gives


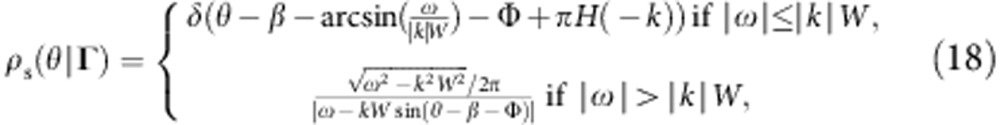


where *H*(−*k*) denotes a Heaviside function. As can be seen, the oscillators with |*ω*|<|*k*|*W* (in a rotating frame) are frozen around the positions determined by *β*, *ω*, *k* and the weighted mean field *Y*=*We*^*i*Φ^, while the others are incoherent.

It should be noted that, in addition to *a*_s_(**Γ**) given by equation (17), there exists one more stationary solution of equation (7): the same as equation (17), but with a minus before the 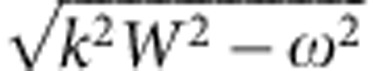 for |*ω*|≤|*k*|*W*; however, this solution is never realized in reality because it corresponds to the unstable position on the phase circle, as can be seen by recovering the respective CPDF (which will be the same as equation (18), but with *θ*→*θ*+*π* for |*ω*|≤|*k*|*W*).

### Self-consistency and stability conditions

While the solution (18) gives a qualitative picture of how the phases are distributed in the stationary regime, one cannot infer directly from *ρ*_s_(*θ*|**Γ**) the stability of such a configuration or the macroscopic parameters *W*,Ω for which it can be realized. To find the latter, we first note that the equation (17) was derived from equation (7) only, without taking account of equation (8), which it should also satisfy. Therefore, substituting equation (17) into equation (8), one obtains the self-consistency conditions (SCCs)


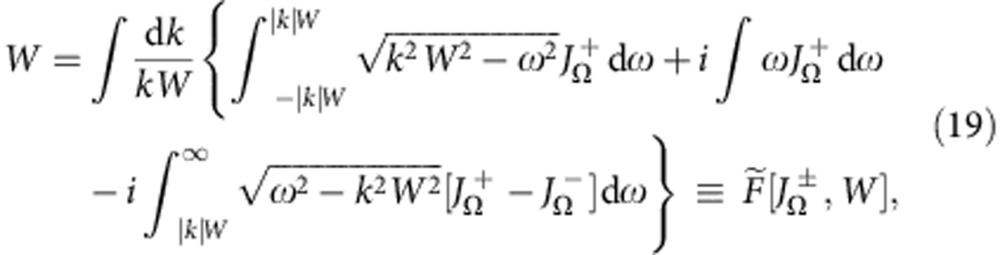


where 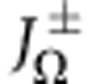 are as defined in equation (12). Taking the real and imaginary parts of equation (19) yields two equations from which the SS parameters *W*,Ω can be determined. These can then be used to determine *Z* as well, for which, using equation (17) in equation (9), one gets


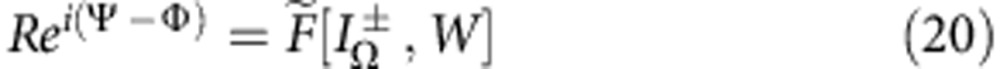


The SS stability, on the other hand, is generally hard to study analytically. For the incoherent state (*W*=0), however, it can be analysed using the approach of ref. 45. Thus, performing a linear stability analysis of (5) above incoherence and using a self-consistency argument (see Methods), one can show that incoherence changes stability when there exists a solution *x* to the equations


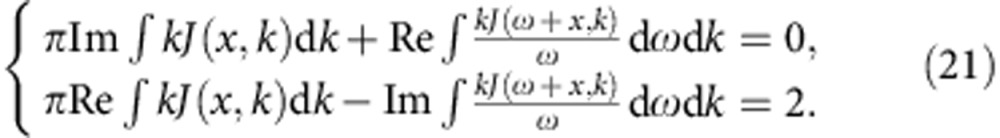


Interestingly, from equations (21) and (12) it follows that in the case *G*(**Γ**)=*G*(*ω*, *q*, *β*, *γ*)*p*(*k*) the stability of incoherence does not depend on a particular form of *p*(*k*), but only on the mean coupling strength 〈*k*〉≡∫*kp*(*k*)d*k*, as noticed previously for a simpler model^10^,^15^. Note also that, if phase shifts *γ* or *β* are present then, with increasing coupling strength, the incoherence can not only lose, but also gain^46^,^47^ stability at the transition points determined from equation (21).

To estimate (at least approximately) the stability of the SSs with *W*>0, one can utilize the approach of ref. 10 and devise the empirical stability conditions (ESCs) which, for the considered model (1), take the form (see Methods)


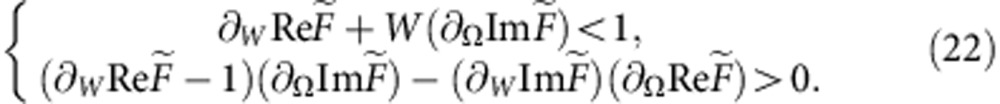


where 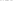 (see equation (19)). Despite being empirical and thus approximate, the ESCs (22) work well in the majority of cases, though not in all, for example, they can fail in the presence of standing waves^10^; their performance also becomes less good when arg[*J*(*ω*,*k*)]≠0. Nevertheless, ESCs seem to be exact if arg[*J*(*ω*,*k*)]=0 and the distribution *G*(**Γ**) is unimodal over *ω*.

### Uncoupled distributions

As discussed above, for the system (1), the parameters of its possible SSs can be found from the SCCs (19) and (20), their stability can be deduced from equation (21) for the incoherent state, and (approximately) from equation (22) for other SSs, while the associated phase distributions are given by equation (18). The corresponding expressions, however, are generally quite complicated, but it turns out that they simplify greatly if we consider the distribution of *q*, *β*, *γ* to be uncorrelated with *ω*, *k*. In what follows, we will therefore assume that


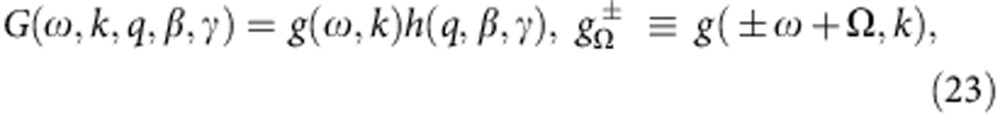


so the effective (*ω*,*k*)-distributions (12) become


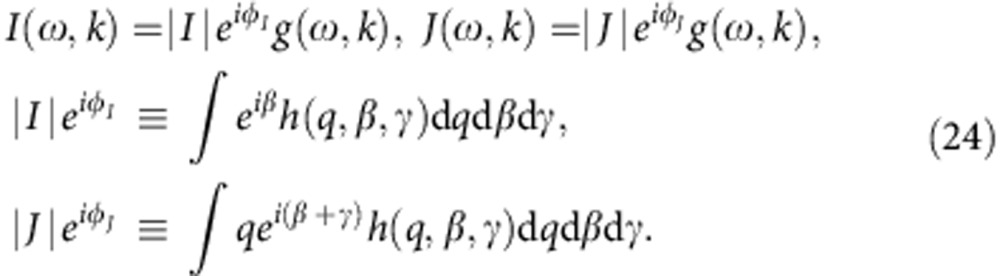


The SCCs (19) then simplify to


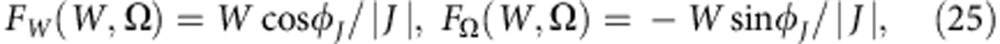


where


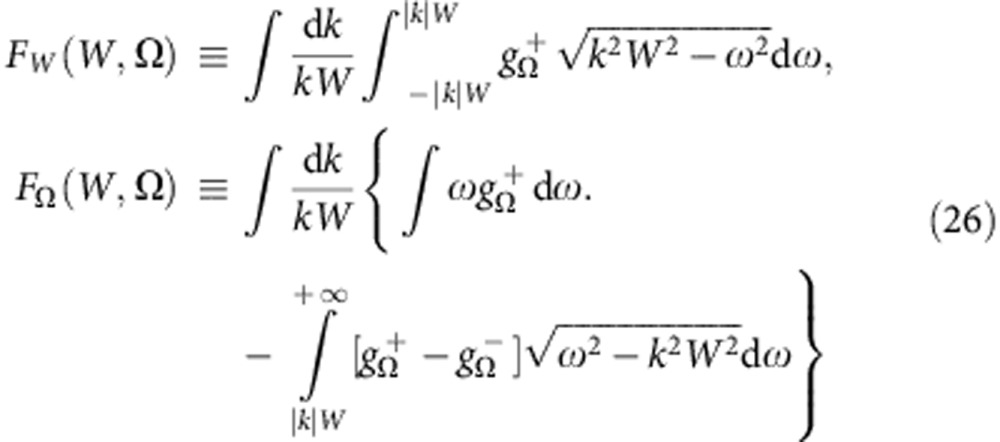


are fully analogous to *F*_*R*,Ω_ in ref. 10; the expression (20) for *R* and Φ−Ψ reduces to


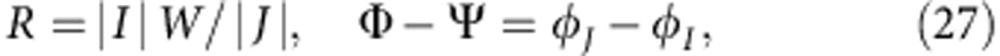


the incoherence stability conditions (21) become


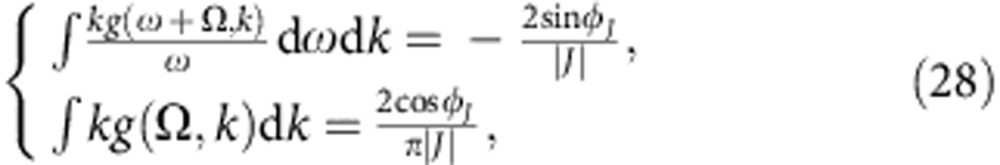


and the ESCs (22) can be simplified using 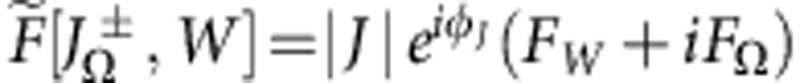.

### System reduction

From equations (25–28) it is evident that, for uncoupled distributions (23), all the macroscopic properties of the SSs are completely characterized by *g*(*ω*, *k*), |*J*| and *ϕ*_*J*_ (see equation (24)), irrespectively of the particular form of *h*(*q*, *β*, *γ*), while |*I*|, *ϕ*_*I*_ serve merely to specify *Z* (27). Instead of (1), therefore, one can consider the system


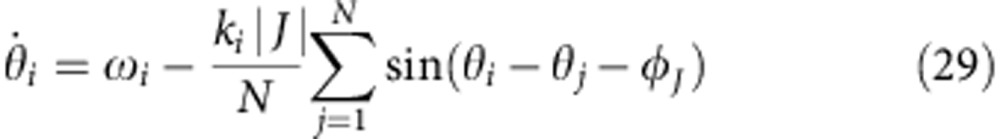


with the same distribution of *ω*_*i*_, *k*_*i*_ defined by *g*(*ω*, *k*). Then each SS of (29) corresponds to an SS of (1), and their parameters are related as


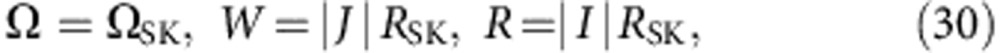


where the subscript SK denotes the states of the model (29); the stability of the corresponding states will also be the same, as follows from equation (28) for incoherence, and from the ESCs (22) and numerical evidence for other SSs.

**Figure 1:**
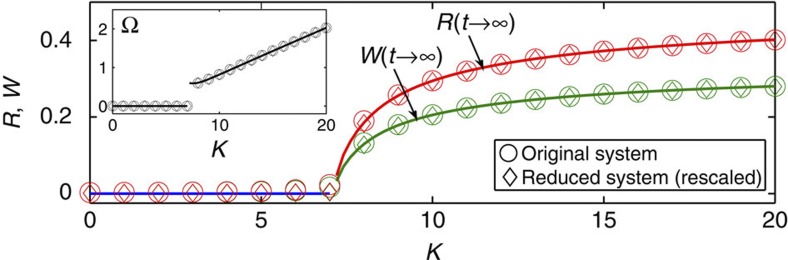
Numerical confirmation of system reduction. The SS parameters *W* (green), *R* (red) and Ω (black), as calculated for the original system (1) (circles) and obtained from the reduced system (29), (30) (diamonds), are shown in dependence on coupling *K*; solid lines correspond to theoretical predictions based on (25–28). The distribution for the original system was: 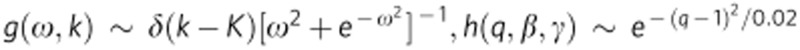

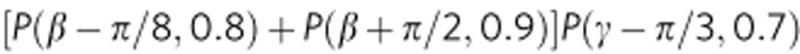
, but the same picture appears for any *h*(*q*, *β*, *γ*) with identical *ϕ*_*J*_, |*J*|, |*I*| (24). The simulations were performed for 500 s, and the values presented are averages over the last 100 s.

Hence, for any distribution of *q*, *β*, *γ* obeying (23), one can reduce the consideration of the steady-state behaviour of (1) to the much simpler Sakaguchi–Kuramoto model (29). This elegant and unexpected result is illustrated in Fig. 1. Interestingly, something similar was noted earlier^21^, but for a significantly less general model than (1). The reduction (30), however, relates only to the macroscopic properties of SSs (*t*→∞), whereas the full evolutions *Z*(*t*),*Y*(*t*) and the microscopic properties of the resultant states cannot be obtained in this way. Note that equation (30) implies *W*≤|*J*|,*R*≤|*I*|, that is, the distribution of *q*, *β*, *γ* imposes an upper bound on the SSs’ mean field strengths, which they cannot exceed however strong the coupling is.

### Definition of glassy states

Ordinary glass^30^ is a state of matter which, in contrast to the liquid state, is solid; but, in contrast to the crystalline state, has a structure without any long-range translational order. States that are in some sense similar were reported in several other systems^26^,^27^,^28^,^29^, but whether or not there can exist glassy analogues in networks of coupled oscillators—oscillator glasses—has remained unclear^25^,^31^,^32^,^33^,^34^,^35^,^36^.

However, a very basic question is how to define an oscillator glass, that is, what properties the system should possess in order to qualify for this title. Obviously, one should draw an analogy with known glassy states in order to illuminate this issue. Thus, the defining feature of the structural glasses^30^—frozen disorder—can be specified as: (i) the particles, for example atoms, lack any long-range translational order, similarly to the liquid state; (ii) the particles are frozen, that is, do not move with respect to each other, similarly to the solid state. Later, states with frozen spins lacking long-range orientational order were discovered in spin systems, and by analogy were called spin glasses^26^,^27^. The canonical spin glasses, in addition to properties (i) and (ii), are characterized by: (iii) a significantly redundant ground state, so that the same macroscopic behaviour can be realized by many microscopic spin configurations, not related to each other by any simple symmetry transformation; (iv) frustration, that is, no spin configuration can simultaneously satisfy all energy bounds; (v) slow non-exponential relaxation of the order parameter (magnetization for spin systems), as well as other exotic dynamical features.

Relating the phases *θ*_*i*_ of the oscillators to the positions of the particles in space, or the spin orientations, the analogies of the above properties for populations of oscillators will be (all for *t*→∞)


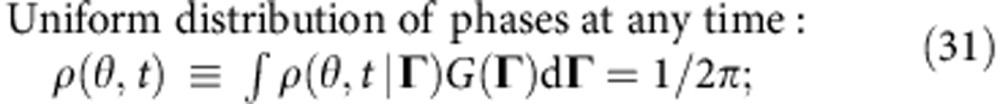



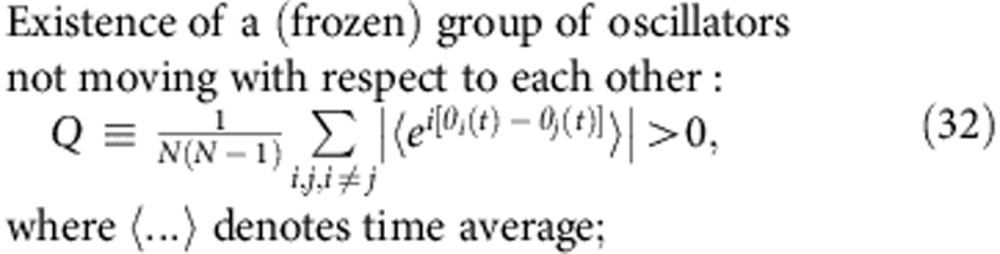



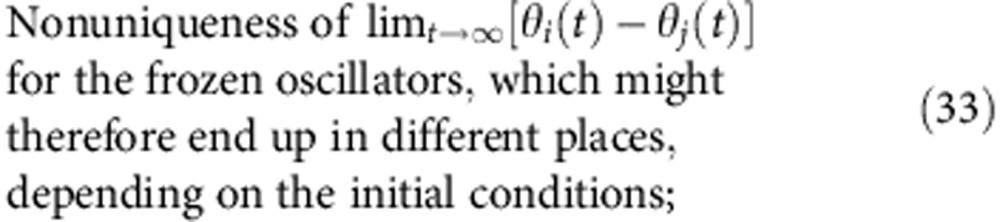



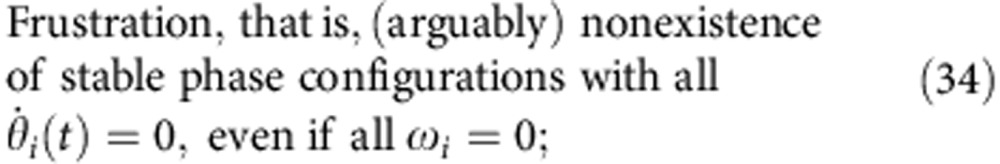



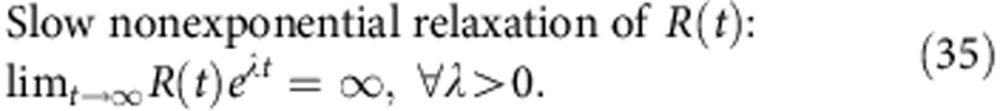


Generally, it is quite a subtle issue to decide what is a ‘true’ oscillator glass, that is, should it satisfy all the criteria (31–35), or only some of them? Thus, the state reported by Daido^25^ has properties (31), (34) and (35), but not (32) and (33), since the oscillators there, although adjusting their mean phase velocities to some extent, perform a diffusive motion and thus, are never frozen (*Q*=0). This state, therefore, bears more analogy to spin glasses than to structural glasses.

Here, we utilize a different approach, considering state to be glassy if it satisfies the first two criteria (31), (32). To avoid possible terminological issues, we refer to such states as quasi-glassy. They are thus defined as the states with a uniform distribution of phases *θ*_*i*_ (31), indistinguishable from incoherence but where, in contrast to the latter, some macroscopic portion of oscillators are frequency-locked (32), that is, have equal phase velocities 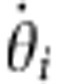.

It should be noted that the condition for the absence of phase-locking is sometimes taken as *R*=0. However, although being implied by equation (31), it is not as rigorous as the latter. Thus, there might be many configurations for which *R*=0 but where the oscillators actually adjust their phases. Examples include two phase-locked populations of the same size, which are in the anti-phase with each other. States with *R*=0 satisfying condition (32), but not condition (31), will be called spurious glassy, according to ref. 31 where they were first discovered. All SSs with *R*>0, including the usual synchronized states, *π*-states^19^,^20^ and travelling waves, will be classified as coherent.

As a simplified picture, the distinctions between the states can be understood in terms of a group of people doing cyclical exercises, each with their own tempo and other parameters. The incoherent state is when everyone proceeds independently; the coherent state is when they all move synchronously, at any given time having similar poses; spurious glassy is when they exercise at the same tempo but remain pairwise in the opposite poses, and quasi-glassy is when they adjust their tempos, but always remain in the independent random poses, thus representing a kind of synchronous disorder.

### Realization of glassy states

Considering model (1) with the uncoupled distribution (23), it can be shown (see Methods) that if the marginal distribution of *β* is uniform and |*J*|>0, then all SSs except incoherence satisfy equations (31) and (32). Therefore, the quasi-glassy states appear when


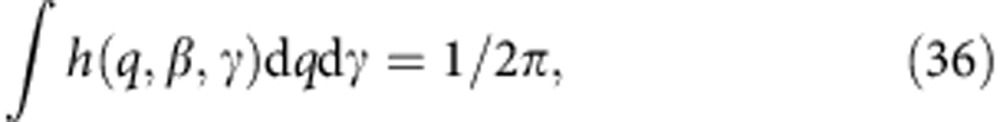



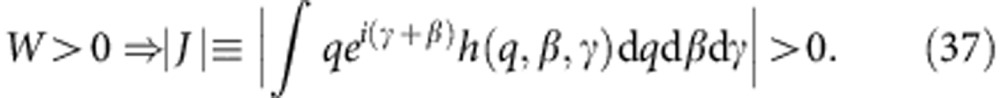


Representing *h*(*q*, *β*, *γ*)≡*h*_1_(*β*)*h*_2_(*q*, *γ*|*β*), equation (36) becomes *h*_1_(*β*)=1/2*π*. Thus, to satisfy also condition (37), the distribution of *q*, *γ* should be specifically correlated with *β*. The simplest examples are *h*(*q*, *γ*|*β*)=*δ*(*q*−*q*_0_)*P*(*γ*+*β*,*r*) and *h*(*q*, *γ*|*β*)=*L*(*q*−*q*_0_cos*β*, Δ)*P*(*γ*−*γ*_0_, *r*).

For spurious glassy states, the condition (36) is not satisfied, but *R*=0, which in the present case, is equivalent to |*I*|≡|∫*e*^*iβ*^*h*_1_(*β*)d*β*|=0, as can be seen from equation (27). There are many possible *h*_1_(*β*) satisfying this condition, for example, *h*_1_(*β*)=*P*(*β*−*β*_0_,*r*)+*P*(*β*−*β*_0_−*π*,*r*).

**Figure 2:**
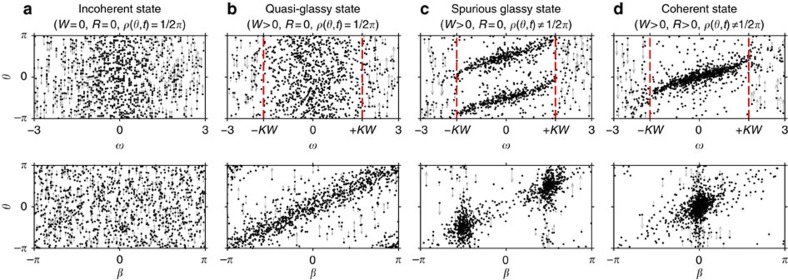
Different kinds of states. The states that can appear in the model (1), illustrated by snapshots of positions in the *θ*, *ω* (upper panel) and *θ*, *β* (bottom panel) planes of (the same) 1,000 randomly selected oscillators (out of *N*=25,600). Tiny vertical arrows show the oscillators movements during 0.25 s (for a dynamic version of the figure see Supplementary Movie 1). Red-dashed lines show boundaries between the clusters and incoherent populations. In all cases, *g*(*ω*, *k*)=*L*(*ω*, 1)*δ*(*k*−*K*), *h*_2_(*q*,*γ*|*β*)=*δ*(*q*−1)*δ*(*γ*+*β*), and (**a**) *K*=1, *h*_1_(*β*)=1/2*π*; (**b**) *K*=3, *h*_1_(*β*)=1/2*π*; (**c**) *K*=3, *h*_1_(*β*)=(1/2)[*P*(*β*−*π*/2,0.8)+*P*(*β*+*π*/2,0.8)]; (**d**) *K*=3, *h*_1_(*β*)=*P*(*β*, 0.8). The pictures will be the same for any *h*_2_(*q*, *γ*|*β*) satisfying |*J*|=1, *ϕ*_*J*_=0 in each case. All states have Ω=0 and are presented in the natural frame (〈*ω*〉=0); depending on the distribution *G*(**Γ**) of parameters in (1), similar states may also appear in a rotating frames (Ω≠0), corresponding to quasi-glassy, spurious glassy or coherent travelling waves.

Examples of the four types of states occurring in the model (1) are shown in Fig. 2, presented in the natural frame where the population does not move as a whole (〈*ω*〉=0). In the (*θ*,*ω*)-plane, the only difference between the quasi-glassy state and incoherence is that, in the former, phases within the glassy cluster (|*ω*|<|*k*|*W*) are frozen around random angles *β* (static disorder), as seen in (*θ*, *β*)-plane; this is in contrast to their asynchronous movement (dynamic disorder), observed for incoherence. Drawing an analogy between the oscillators’ phases and the particles’ positions in space, the incoherent state of the system (1) can be related to a liquid or gaseous state of matter; coherent to the crystalline state; spurious glassy to a crystal with a specific structure; and quasi-glassy to a glass.

As discussed previously, the model (1) can be reduced to a form without *β* (10) by a change of variables. Thus, any quasi-glassy or spurious glassy state in terms of *θ*_*i*_ (1) can be transformed to a coherent state in terms of 
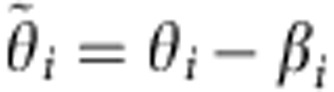
. Contrariwise, glassy states of the system (1) can always be viewed as coherent states but in a specifically disordered coordinate system, being therefore redundant in a strict mathematical sense. In practice, however, the coordinates are chosen based on their physical meaning, so that these states are realizable when the actual interaction between the oscillators contains phase shifts *β*, which can be achieved, for example, in a real systems with programmable coupling^48^,^49^. A similar situation occurs for the Mattis spin glass^50^: it can be transformed to a ferromagnetic state by change of variables^26^,^27^. Nonetheless, the Mattis model found a number of applications^51^,^52^,^53^ and proved to be a useful starting point in studies of spin glasses. Note that, combining the results of the present work with those of refs 22, 32, 33, 54, one can modify the model (1) to observe states exhibiting most of the glassy properties (31–35) (see Methods).

### Relaxation dynamics

Up to now, we have concentrated mainly on the behaviour of the system (1) in the asymptotic limit *t*→∞, restricting our consideration to a set of its possible SSs. The relaxation to these states is determined by the parameter distribution and can take various forms. It is hard to analyse in general, but for particular *G*(**Γ**) the full-time evolutions *Y*(*t*),*Z*(*t*) can be obtained analytically by using the OA-reduction procedure^41^, which can be extended to incorporate the distributed phase shifts. Thus, consider the distribution


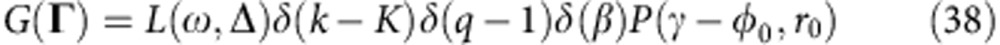


In this case, *a*(**Γ**,*t*) can be analytically continued inside the unit circle of *z*_*γ*_=*e*^*iγ*^, as the condition (15) is satisfied there. Hence, in equations (8) and (9), one can integrate over *γ* by changing the integration to the unit circle of *z*_*γ*_ and taking the residue at the pole 
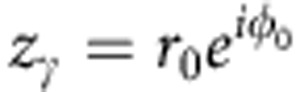
 of *P*(*γ*−*ϕ*_0_,*r*_0_) (see equation (11)). The integration over *ω* is performed in the usual way^41^, that is, by taking the residue at *ω*=*i*Δ. Then, from equations (8) and (9) one obtains 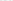, 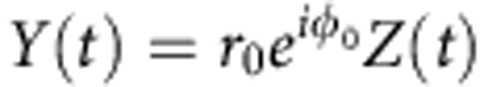. Substituting this into equation (6) and solving the resultant equations, one gets finally


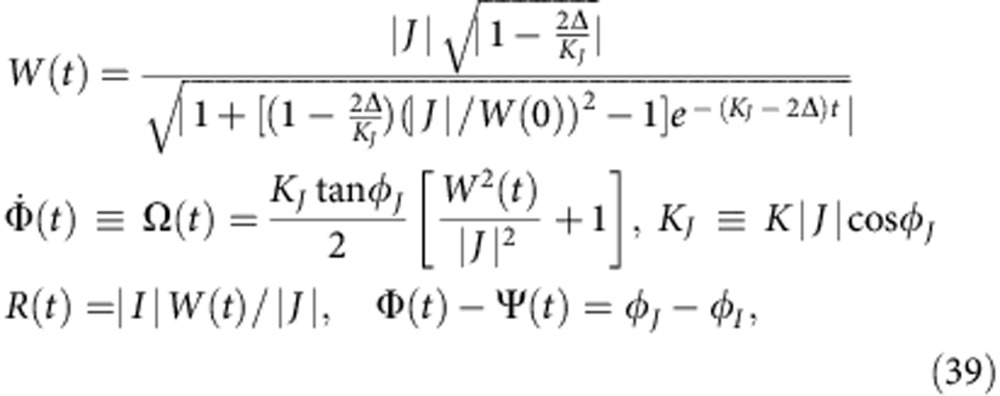


where we have represented all in terms of *I*, *J* (see equation (24)), which in the present case are 
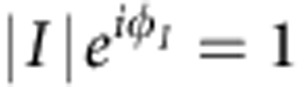
, 
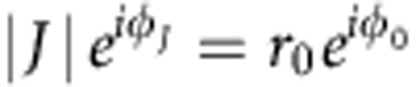
. Obviously, a similar procedure to that outlined here can be applied if the distribution of *γ* has a few poles inside the unit circle.

Now consider the KM (1) with distributed *β*:


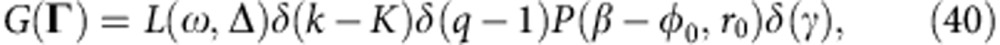


in which case, one has *Z*(*t*)=*Y*(*t*) by definition (2). By the change of variables 
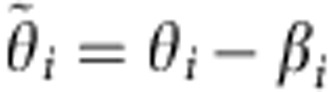
, the present case can be reduced to the already-studied KM with distributed *γ* (see equation (38)). Then, because 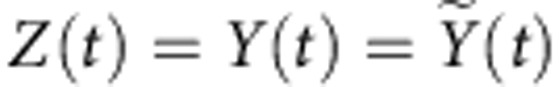 (see discussion of equation (10)), the macroscopic parameters should still evolve according to equation (39), although one should now use 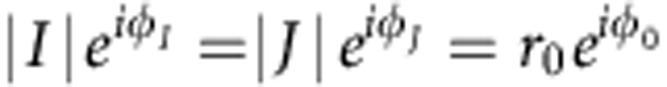 in the latter. The same result may be obtained by applying the OA-reduction, with the integrals (8) and (9) over *β* being evaluated by taking residues inside the unit circle of *e*^*iβ*^, similarly to what was done in the previous case.

**Figure 3:**
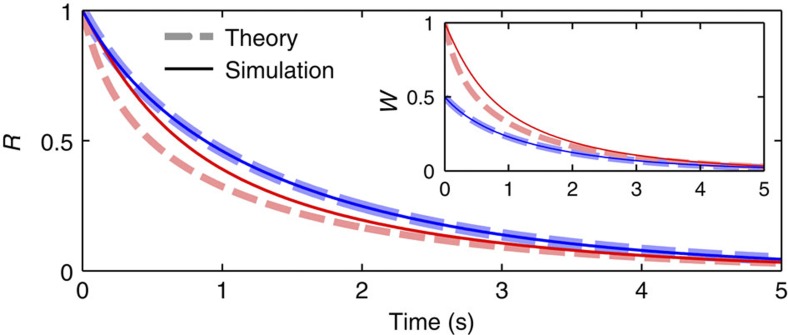
Theoretical and numerical system behavior. The main plot and inset display time evolutions of *R*(*t*) and *W*(*t*), respectively. Dashed lines show the behaviour predicted by (39), while solid lines correspond to the results of numerical simulations: blue—distributed *γ* (38), red—distributed *β* (40). In (38) and (40), we used *ϕ*_0_=*π*/6, *r*_0_=0.5.

However, the behaviour predicted by equation (39), and the actual behaviour of *Z*(*t*),*Y*(*t*), agree only for distributed *γ*, but not for distributed *β*, as demonstrated in Fig. 3. The reasons for this are, first, that one cannot continue *a*(**Γ**,*t*) inside the unit circle of *β*, as (15) is not satisfied there, which makes OA-reduction impossible when *β* is distributed. Second, the evolution of *Y*(*t*),*Z*(*t*) for (40) cannot be obtained from the system of 
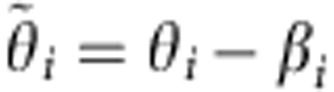
, at least if starting from the standard initial conditions (16), as assumed here. This is because such transformation introduces a correlation of the initial phases with system parameters. For example, if all *θ*_*i*_(0)=0, then one will have 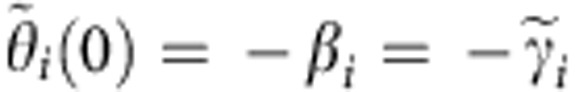, so that 
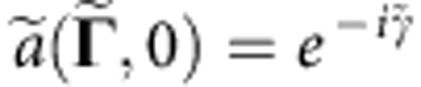
 and the OA-reduction cannot be applied because now (14) is not satisfied. Therefore, although the cases (38) and (40) can be transformed into each other by changes of variables, the time evolution for distributed *β* appears to be much more complex than for distributed *γ*, and cannot be obtained in a simple form.

### Super-relaxation

Astonishingly, for a class of distributions *G*(**Γ**), and a very large family of initial configurations, the oscillators do not feel any interaction at all while relaxing to incoherence. This behaviour occurs when the following three conditions are satisfied:


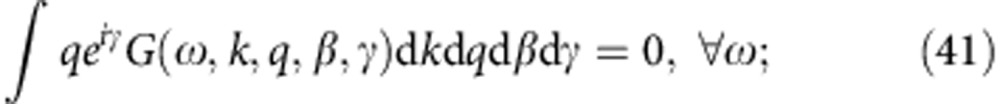



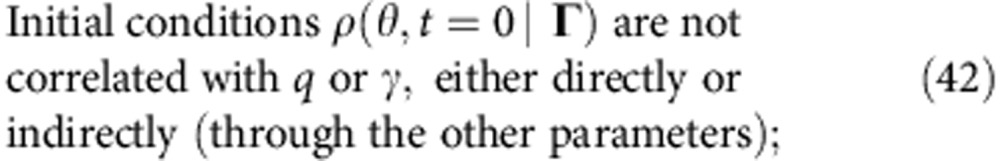



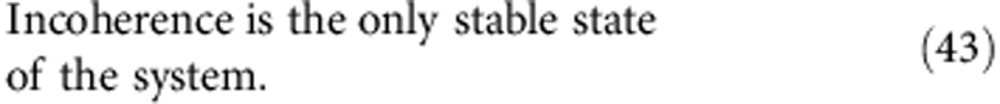


Thus, conditions (41) and (42) imply *W*(0)=∫*e*^*iθ*^*ρ*(*θ*,0|**Γ**)*qe*^*iγ*^*G*(**Γ**)d*θ*d**Γ**=0. Based on numerical evidence, the weighted mean field then stays at zero during the whole evolution *W*(*t*)=0, leading to an effective disappearance of interaction between the oscillators, as follows from equation (3). As a result, the oscillators evolve freely (
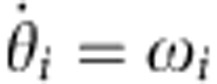
) and so the relaxation (both its rate and form) depends only on the marginal distribution of *ω*:


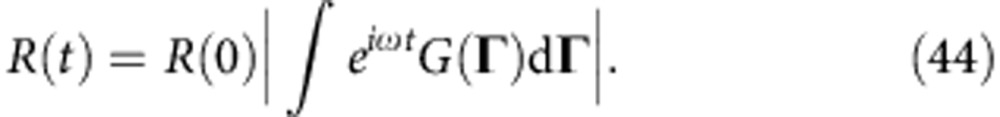


**Figure 4:**
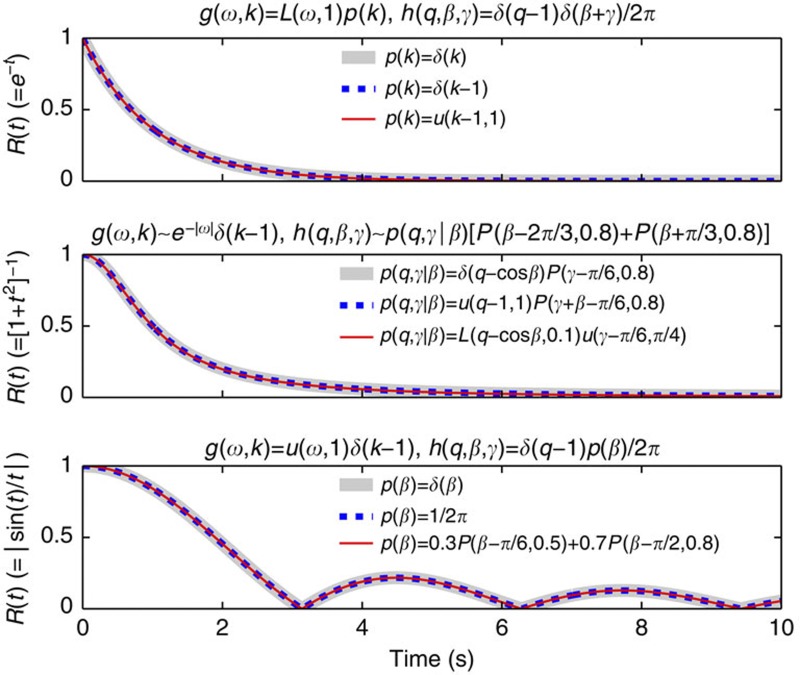
Examples of super-relaxation. Interaction-independent relaxation of the order parameter to incoherence for different parameter distributions (all satisfying (41)), which are indicated in the figure; *u*(*x*, *b*) denotes uniform distribution of *x* in [−*b*, *b*], while *L*(*x*, Δ) and *P*(*x*, *r*) are defined in (11).

This phenomenon, which we refer to as super-relaxation, is illustrated in Fig. 4. Note that for uncoupled distributions, equation (23), the condition (41) reduces to ∫*qe*^*iγ*^*h*(*q*, *β*, *γ*)d*q*d*β*d*γ*=0.

**Figure 5:**
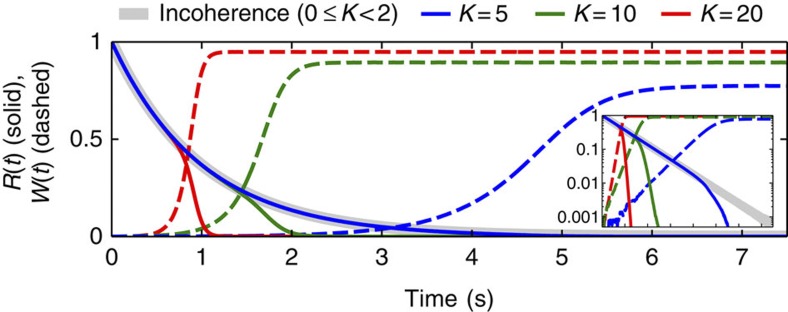
Two-stage relaxation to quasi-glassy states. Time evolutions of *R*(*t*) (solid) and *W*(*t*) (dashed) from the initial conditions *R*(0)=1 to quasi-glassy states (*g*(*ω*, *k*)=*L*(*ω*, 1)*δ*(*k*−*K*), *h*(*q*, *β*, *γ*)=*δ*(*q*−1)*δ*(*β*+*γ*)/2*π*) for different constant couplings *k*_*i*_=*K*. The inset shows the results on a logarithmic ordinate scale.

The claim that *W*(*t*)=0 if all the conditions (41), (42) and (43) are satisfied can be proven rigorously for particular distributions, for example, *G*(**Γ**)=*G*(*ω*, *k*, *q*, *β*+*γ*)/2*π* (see Methods), though we were unable to prove it in general; based on simulations, however, it seems to be satisfied in most (if not all) cases. Thus, it is clear that the system always has a solution with *W*(*t*)=0 if conditions (41) and (42) are fulfilled: in this case, to have *W*(*t*)>0, the distribution *ρ*(*θ*, *t*|**Γ**) should acquire a particular dependence on the system parameters; but evolving according to equation (5) with *W*(*t*)=0, it can become dependent only on ω, which in turn cannot increase *W*(*t*) above zero if condition (41) holds. The stability of this solution is rather hard to prove, but it appears to be stable if the incoherence is the only stable state (condition (43)). Nevertheless, even if *W*(*t*) is not exactly zero, below some relatively high threshold its value seems to have a negligible effect on the dynamics of *R*(*t*), as will be seen in Fig. 5 below.

Super-relaxation can appear only if the final state is incoherence (*W*=0), while relaxation to SSs with *W*>0 will generally be coupling dependent, even if conditions (41) and (42) are satisfied. In the latter case, relaxation occurs in two stages as shown in Fig. 5 for the example of quasi-glassy states. The phases first begin to disorder in the same way as for incoherence, but are slowly entrained while passing their equilibrium positions. When the field of the entrained oscillators, characterized by *W*, becomes strong enough, they begin to force the unentrained ones to take their positions, so the relaxation switches to a faster, coupling-dependent regime. This switch occurs sooner for stronger coupling.

## Discussion

We have generalized our earlier approach^10^ to make it applicable to the more general KM (1) so that, using equations (19–22), one can immediately obtain a macroscopic characteristics of possible SSs. The generalized KM (1) encompasses a variety of KM variants studied earlier^10^,^13^,^14^,^15^,^19^,^21^,^24^, allowing one readily to reproduce and extend many of the previous results. Remarkably, the steady-state behaviour of (1) with any distribution *h*(*q*,*β*,*γ*) (see equation (23)) can be obtained from the simple Sakaguchi–Kuramoto model (29), (30). It should be noted, however, that (17–22) describe only SSs, being inapplicable to inherently non-stationary solutions such as standing waves^55^ or oscillating *π*-states^44^. Note also that most expressions can straightforwardly be extended to the case *G*(**Γ**)→*G*(**Γ**,*R*,*W*) (see Methods); for examples when this might be relevant see refs 56, 57, 58, 59.

Most interestingly, we have found, that the model (1) can exhibit exotic behaviour, such as glassy states and super-relaxation, thereby opening new horizons for KM-related investigations, both theoretical and practical. These discoveries have a far-reaching implications. For example, it should now be possible to create, observe and study glassy behaviour in real systems of coupled oscillators, where a variety of novel phenomena may be anticipated. As one possible application, if some physical quantity can be associated with the weighted mean field *W* (equation (2)) in laser arrays^5^ described by the KM, it might be possible to construct a laser exhibiting zero intensity (*R*=0) but for which the other effects are nonvanishing (*W*>0); similar considerations apply to a wide range of different KM applications. Furthermore, the phenomenon of super-relaxation might be used to design systems whose dynamics remains highly stable in the face of different perturbations and parameter changes.

## Methods

### Numerical simulations

Except where otherwise specified, all simulations were performed by integrating the full system equation (1) using a 6th order Runge–Kutta method with a time step of 0.01 s. The number of oscillators used was *N*=25,600 for Fig. 2, *N*=10^5^ for Fig. 1 and *N*=10^6^ for all other cases. Additional details are provided in figure captions.

### Parameter normalization

Since the model (1) is invariant under (*k*_*i*_, *β*_*i*_)→(−*k*_*i*_, *β*_*i*_+*π*) or (*q*_*i*_, *γ*_*i*_)→(−*q*_*i*_, *γ*_*i*_+*π*) or rescalings (*k*_*i*_, *q*_*i*_)→(*k*_*i*_/*r*, *rq*_*i*_), there exist some ambiguity in the parameter definitions. To remove this ambiguity, one can fix the normalization of *q*_*i*_ and specify a rule for choosing the sign of *k*_*i*_, *q*_*i*_; but different choices might be preferred in different situations. For example, the normalization ∑*q*_*i*_=1 is inapplicable when *q*_*i*_=cos(*γ*_*i*_) with *γ*_*i*_ being uniform; ∑ |*q*_*i*_|=1, on the other hand, fails for a Lorenzian distribution of *q*_*i*_. The most universal choice seems to be ∫|∫*qe*^*i*(*β*+*γ*)^*G*(**Γ**)d*q*d*β*d*γ*|d*ω*d*k*=1, which additionally assures *W*≤1. However, when *q*_*i*_=1 and *β*_*i*_ are distributed (so *Y*=*Z*), this normalization will lead to a rescaling of *q*_*i*_ (so *Y*≠*Z*), which might be inconvenient. Therefore, because the normalization can be fixed at any time, we retain the associated ambiguity in order to preserve generality. Note that *W* (equation (2)) but not *R* and not |*k*|*W*, changes under the (*k*,*q*)-rescalings and thus can be higher than unity.

### Phase distribution

The distribution *P*(*ϕ*, *r*), equation (11), represents a Poisson kernel for the unit disc and is very common for the KM. For example, it can be shown that the OA-ansatz (equation (6)) can be rewritten^43^ as *ρ*(*θ*, *t*|**Γ**)=*P*(*θ*−arg[*a*(**Γ**, *t*)], |*a*(**Γ**, *t*)|). Because *P*(*ϕ*, *r*) has one pole inside and one outside the unit circle of *z*_*ϕ*_≡*e*^*iφ*^, it is very convenient in relation to analytic derivations, for example, one has 
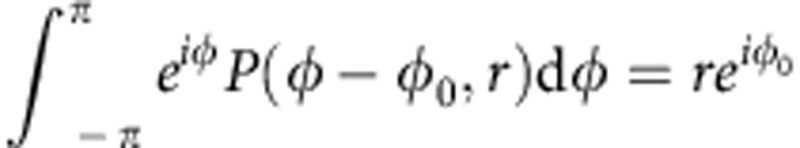
. To simulate this distribution, one sets 
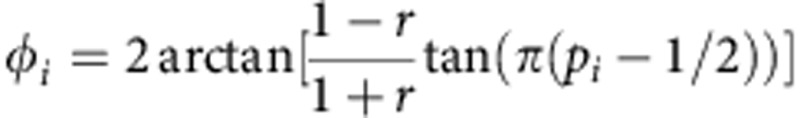
, where *p*_*i*_∈[0,1] are uniformly distributed random numbers.

### Derivation of the incoherence stability conditions

To perform a linear stability analysis of incoherence, we examine the maximum growth rate for small perturbation *εη*(*θ*, *t*|**Γ**) added to the incoherent solution *ρ*(*θ*, *t*|**Γ**)=1/2*π*. Following ref. 45, this perturbation is expressed as


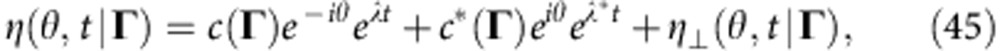


where the Fourier expansion of *η*_⊥_(*θ*, *t*|**Γ**) contains only terms *e*^*inθ*^ with |*n*|>1 (so that ∫(*e*^±*iθ*^)*η*_⊥_(*θ*, *t*|**Γ**)*dθ*=0). Substituting *ρ*(*θ*,*t*|**Γ**)=1/2*π*+*εη*(*θ*,*t*|**Γ**) and (45) into (5), and collecting the terms proportional to *e*^−*iθ*^, to the first order in *ε* one obtains


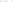


Considering the part ~*e*^*iθ*^ of equation (5), one will get the complex conjugate of equation (46), while for terms ~*e*^*inθ*^ with |*n*|>1 the growth rate of the associated perturbations ∂_*t*_[*εη*_⊥_(*θ*,*t*|**Γ**)] can be shown to be O(*ε*^2^), so that they are of no interest for linear analysis.

From (46) it follows that *c*(**Γ**)=(*ke*^*iβ*^*Y*(*t*)*e*^−*λt*^)/(2*ε*(*λ*−*iω*)). Substituting this into *Y*(*t*)=∫*e*^*iθ*^*ρ*(*θ*,*t*|**Γ**)*qe*^*iγ*^*G*(**Γ**)d*θ*d**Γ**=*εe*^*λt*^∫*qe*^*iγ*^*c*(**Γ**)*G*(**Γ**)d**Γ** and dividing both sides by *Y*(*t*)/2, one self-consistently obtains


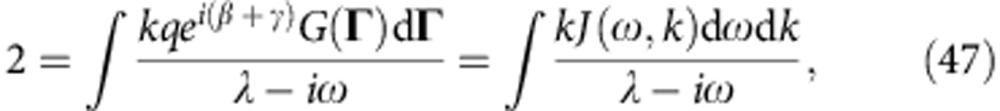


where we have used the definition of *J*(*ω*,*k*) (see equation (12)). The incoherence changes stability when Re[*λ*] crosses zero. Hence, denoting *λ*=*λ*_r_+*iλ*_i_, taking the limit *λ*_r_→0 in equation (47) and using 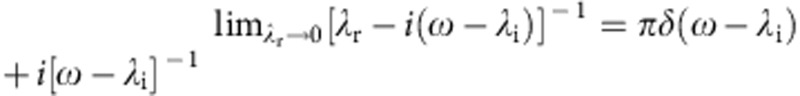, one obtains (21) with *x*=*λ*_i_.

### Derivation of the ESCs

The ESCs (22) cannot be derived rigorously but, rather, are based on empirical assumptions. Thus, we first assume that the perturbations to the SS obey


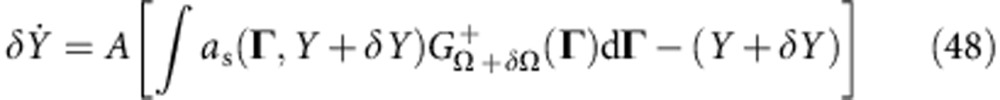


where *δY*=(*δW*+*iWδ*Φ)*e*^*i*Φ^ and *δ*Ω are the deviations of the SS parameters from their stationary values *Y* and Ω, respectively; *A* is a constant, 
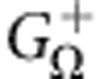
 is defined in (11) and *a*_s_(**Γ**,*Y*) is the OA stationary solution (17). The latter can be represented in unified form for all *ω*, *k* as


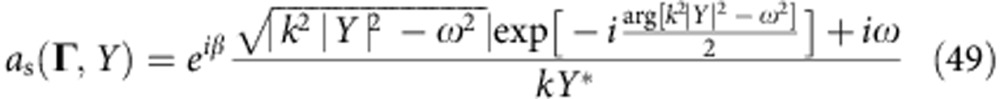


Although equation (48) represents a purely empirical assumption, some of the motivation behind it is that for incoherence it gives the correct transition points (equation (21)), as can be checked by performing a linear stability analysis of equation (48) with *Y*=0.

For *Y*>0, based on trial and error we set *A*>0, *δ*Ω=*W*^2^*δ*Φ in equation (48). Then using equation (49) and retaining in (48) only the terms of the first order in *δW*, *δ*Φ, one obtains


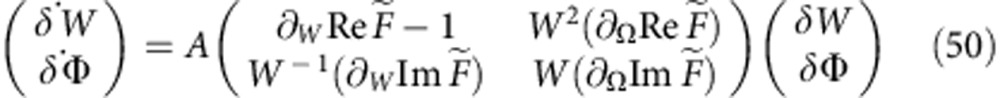


where we have expressed all in terms of 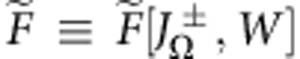 (equation 19). For *A*>0, the system (50) is stable only if the trace and determinant of the corresponding matrix are lower and higher than zero, respectively, which gives (22).

### Glassy conditions for the model considered

For the system equation (1) with the uncoupled distribution, equation (23), it is easy to see that the conditions (31,32) for observing quasi-glassy states can be rewritten as (36,37). Thus, the phases of the oscillators with |*ω*_*i*_|≤|*k*_*i*_|*W* in a stationary regime are frozen at *θ*_*i*_=*β*_*i*_+arcsin(*ω*_*i*_/|*k*_*i*_|*W*)+*πH*(−*k*_*i*_) (all in the rotating frame), as follows from equation (18). Therefore, for states with *W*>0, an absence of phase locking between the oscillators (condition (31)) is equivalent to a uniform distribution of *β*_*i*_ (equation (36)). Next, *W*>0 (37) is the necessary and sufficient condition for (32): necessary because, otherwise, all oscillators have 
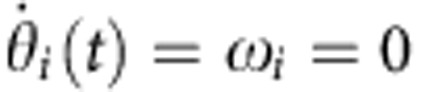
, as implied by equation (3); and sufficient because it establishes frequency locking of the oscillators with |*ω*_*i*_|≤|*k*_*i*_|*W* (since their phases are constant). Finally, as is clear from equation (25), for the uncoupled distributions (23), SSs with *W*>0 can appear only if |*J*|>0, as additionally indicated in equation (37).

### Glassy states exhibiting nearly all glassy properties

One can modify the model (1) to observe glassy states with most of the desired properties (31–35), and not only the first two of them. Thus, frustration (34) can be introduced, for example, by considering the van Hemmen type of interactions^32^,^33^, while the high multiplicity of states (33) can be achieved by using the higher-order coupling^22^,^54^. As an example, it can easily be checked numerically that a state satisfying conditions (31–34) appears in the system


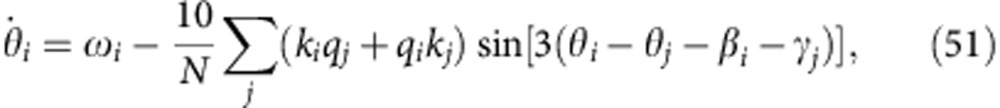


where *k*_*i*_ and *q*_*i*_ are independent random variables taking values ±1 with equal probability, *β*_*i*_=−*γ*_*i*_ are uniformly distributed and the frequencies ω_*i*_ are drawn from *g* (*ω*)~[1+*ω*^2^]^−1^. Regarding the last glassy property (35), it might be possible to satisfy it with an appropriate choice of the parameter distribution, which, to a large extent, determines the relaxation of *R*(*t*). However, most interestingly, it turns out that for systems of the type (51), there exist many configurations with *R*(*t*→∞)>0, and the latter is proportional to the initial *R*(0). Thus, in many cases the phases do not disorder completely, so the basic criterion (31) becomes violated if starting from initial conditions other than incoherence.

### Super-relaxation claim *W*(*t*)=0

In some cases, one can rigorously prove that *W*(*t*) remains zero at all times if the conditions (41), (42) and (43) are fulfilled. Thus, consider the distribution


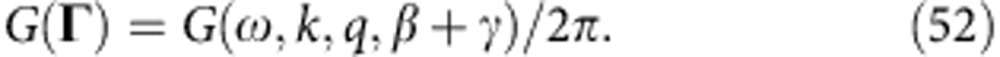


Changing the variables to 
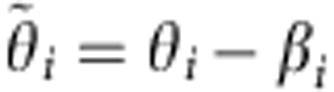
, one obtains the system (10) with 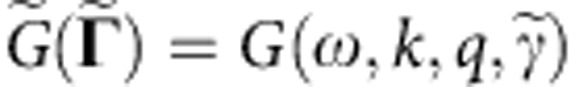, characterized by the same weighted mean field 
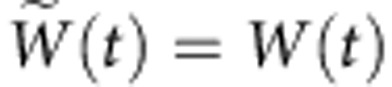
 (see discussion of (10)). Under such a change, however, one subtracts from each *θ*_*i*_ a uniform random variable *β*_*i*_, so that any initial conditions for *θ*_*i*_ satisfying (42) will be mapped to a uniform initial condition for 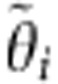; moreover, since *G*(**Γ**) depends only on the sum *γ*+*β*, these initial 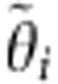 will not be correlated with 
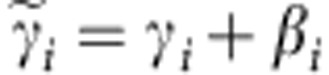
, as would be otherwise. Therefore, one has 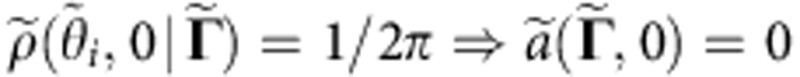. Then, taking into account that 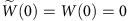, it follows from equations (7) and (8) that 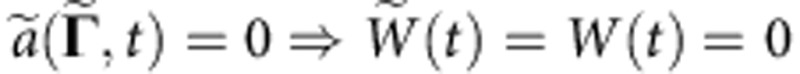. Finally, because the incoherent state for *θ*_*i*_ is stable (condition (43)), it will obviously be stable in terms of 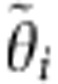 as well, so that the perturbations to the weighted mean field will decay, ensuring that it stays at zero.

### Generalization to *G*(**Γ**)→*G*(**Γ**,*R*,*W*)

In some cases^56^,^57^,^58^,^59^, the parameter distribution might depend on *R* and/or *W*, for example, the coupling can be influenced by the mean field strength *G*(**Γ**, *R*, *W*)~*δ*(*k*−*K*(*R*)). One can easily extend all expressions describing the SSs to such cases. Thus, proceeding in the usual way, the stationary OA solution (17) and the associated phase distribution (18) can be shown to have the same form. Next, the SCCs equations (19) and (20), from which the SS parameters are determined, will also be the same except


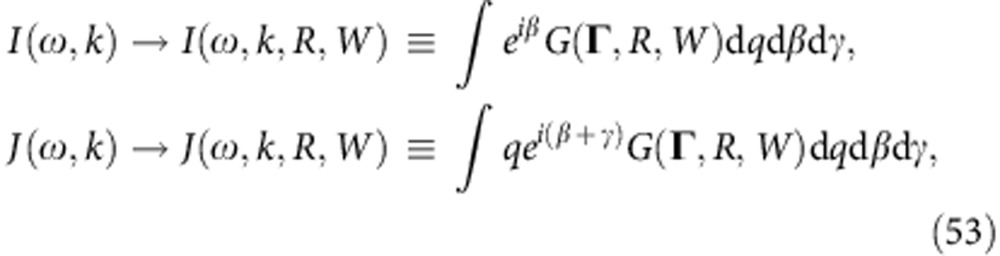


Note, however, that if the parameter distribution depends on *R* (∂_*R*_*G*(**Γ**,*R*,*W*)≠0), and *q* or *γ* are distributed (so that *W*≠*R*), then equations (19) and (20) become coupled and should be solved at the same time to get all the *R*,*W*,Ω,Φ−Ψ. Such situation occurs because, in this case, the effective interaction between the oscillators, although explicitly determined by *W* (3), has additional implicit dependence on *R*; as a result, the dynamics of the model becomes more sophisticated, and its analysis appears to be an interesting topic for future research.

Assuming that 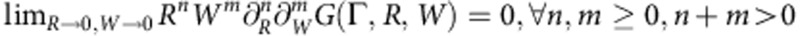, the conditions for incoherence stability can be derived in the usual way and take the same form (21), except that *J*(*ω*, *k*)→*J*(*ω*, *k*, 0, 0) (53). The ESCs (22), on the other hand, cannot so easily be generalized to field-dependent distributions, at least if ∂_*R*_*G*(**Γ**, *R*, *W*)≠0 and *W*≠*R*; otherwise, that is, when *G*(**Γ**)→*G*(**Γ**, *W*), they preserve the original form of equation (22), but with a modified *J* given by equation (53) (though it is not clear how well they work in this case). Having obtained the general equations, their simplification for the uncoupled distributions *G*(**Γ**, *R*, *W*)=*g*(*ω*, *k*, *R*, *W*)*h*(*q*, *β*, *γ*, *R*, *W*) to analogues of equations (24–28) is straightforward.

## Author contributions

D.I. conceived the research, performed the numerics and drafted the manuscript. All authors discussed the results, drew conclusions and edited the manuscript.

## Additional information

**How to cite this article:** Iatsenko, D. *et al.* Glassy states and super-relaxation in populations of coupled phase oscillators. *Nat. Commun.* 5:4118 doi: 10.1038/ncomms5118 (2014).

## Supplementary Material

Supplementary Movie 1 Dynamic version of Figure 2 in the manuscript, illustrating the different kinds of states that can appear in the model. Each point indicates the position of an individual oscillator in the corresponding parameter space.
